# Supplementary material for: Aromatherapy improves cognitive dysfunction in senescence-accelerated mouse prone 8 by reducing the level of amyloid beta and tau phosphorylation
Source: PLoS One. 2020 Oct 14;15(10):e0240378. doi: 10.1371/journal.pone.0240378 (PMC7556469; doi:10.1371/journal.pone.0240378)

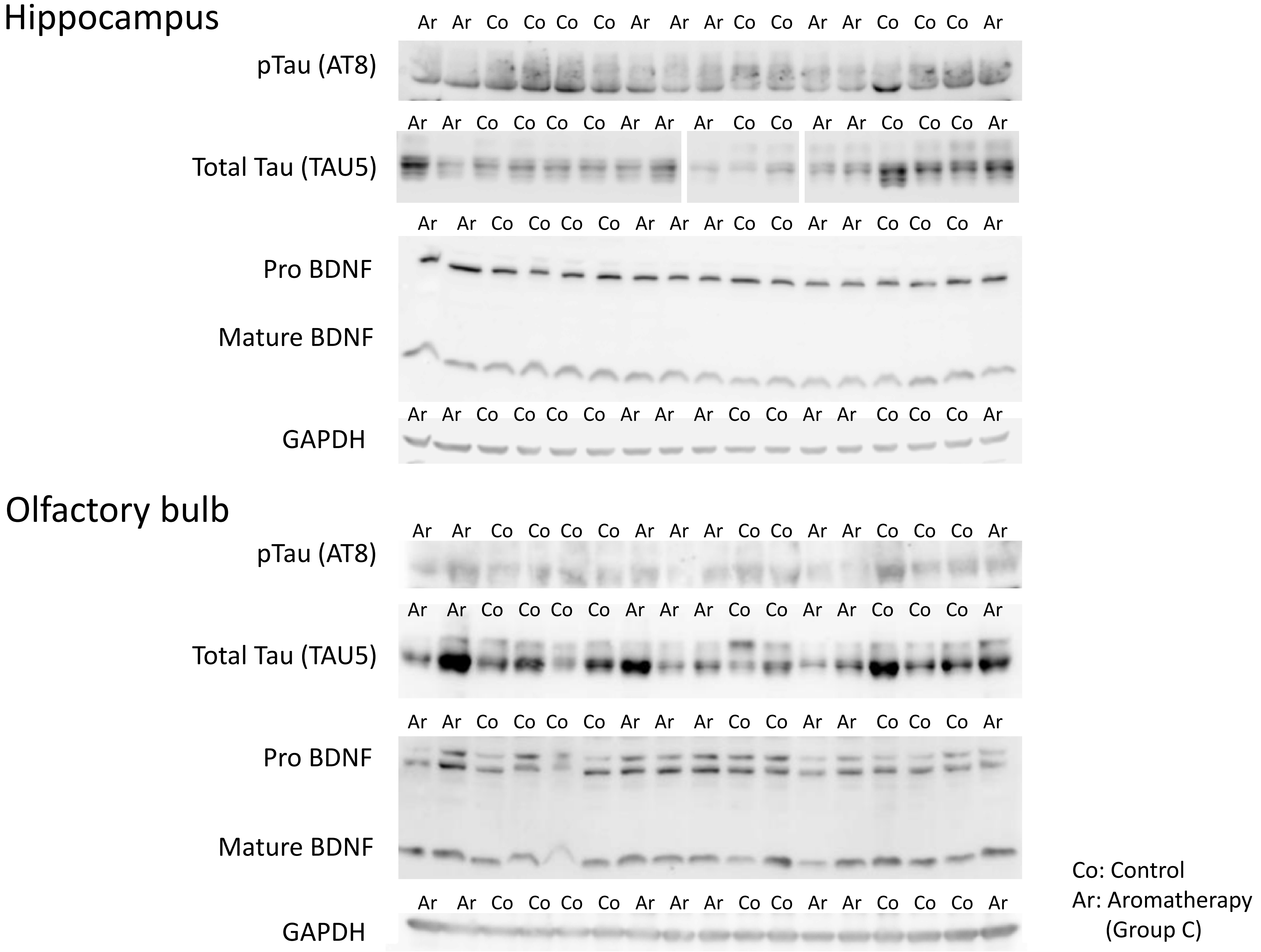
Hippocampus
Ar Ar Co Co Co Co Ar Ar Ar Co Co Ar Ar Co Co Co Ar
pTau (AT8)
Ar Ar Co Co Co Co Ar Ar Ar Co Co Ar Ar Co Co Co Ar
Total Tau (TAU5)
Ar Ar Co Co Co Co Ar Ar Ar Co Co Ar Ar Co Co Co Ar
Pro BDNF
Mature BDNF
Ar Ar Co Co Co Co Ar Ar Ar Co Co Ar Ar Co Co Co Ar
GAPDH
Olfactory bulb
Ar Ar Co Co Co Co Ar Ar Ar Co Co Ar Ar Co Co Co Ar
pTau (AT8)
Ar Ar Co Co Co Co Ar Ar Ar Co Co Ar Ar Co Co Co Ar
Total Tau (TAU5)
Ar Ar Co Co Co Co Ar Ar Ar Co Co Ar Ar Co Co Co Ar
Pro BDNF
Mature BDNF
Ar Ar Co Co Co Co Ar Ar Ar Co Co Ar Ar Co Co Co Ar
GAPDH
Co: Control
Ar: Aromatherapy
(Group C)

Original images

Hippocampus BDNF

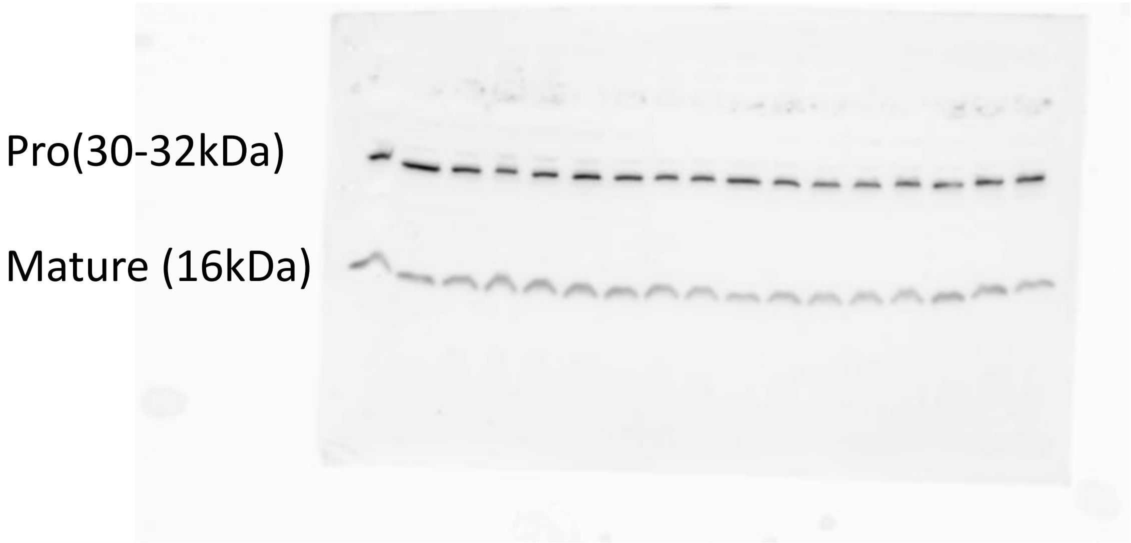

Hippocampus pTau (AT8) and GAPDH (*reblot after BDNF detection shown above)

Long-time exposure

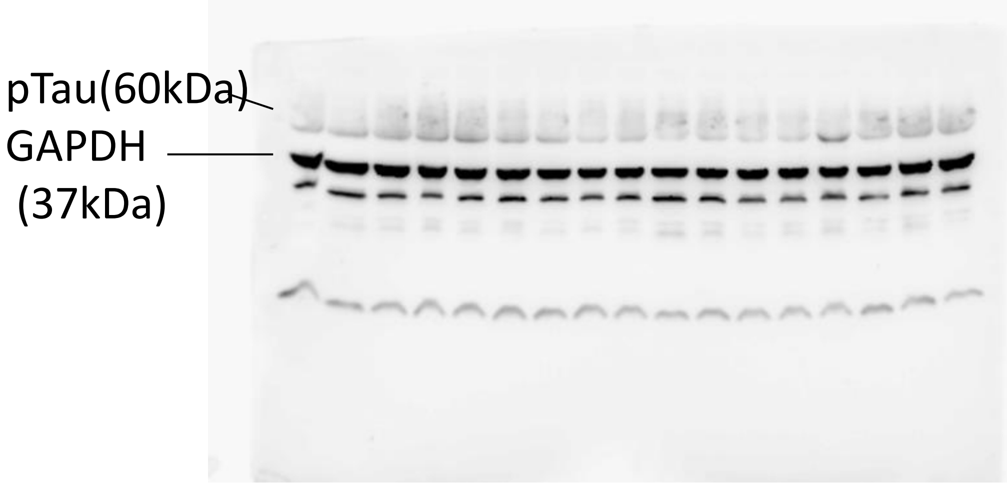

Short-time exposure

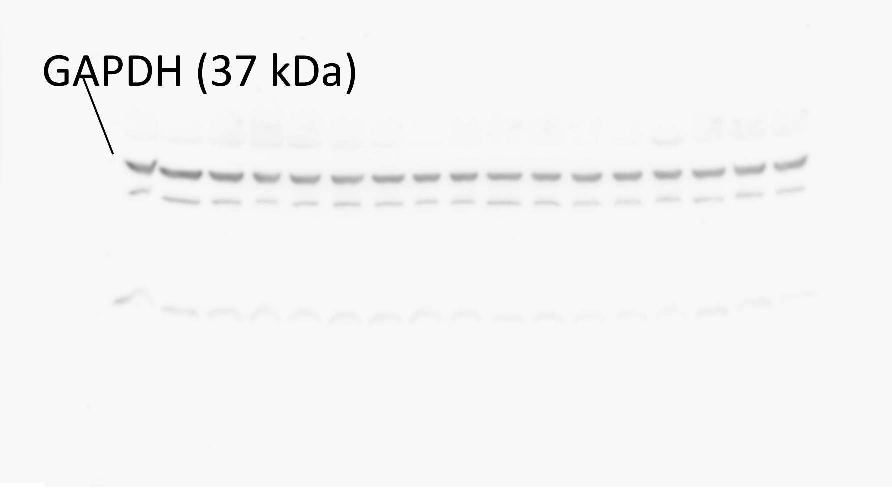

Hippocampus Total tau (*including other samples)

: the samples in this study

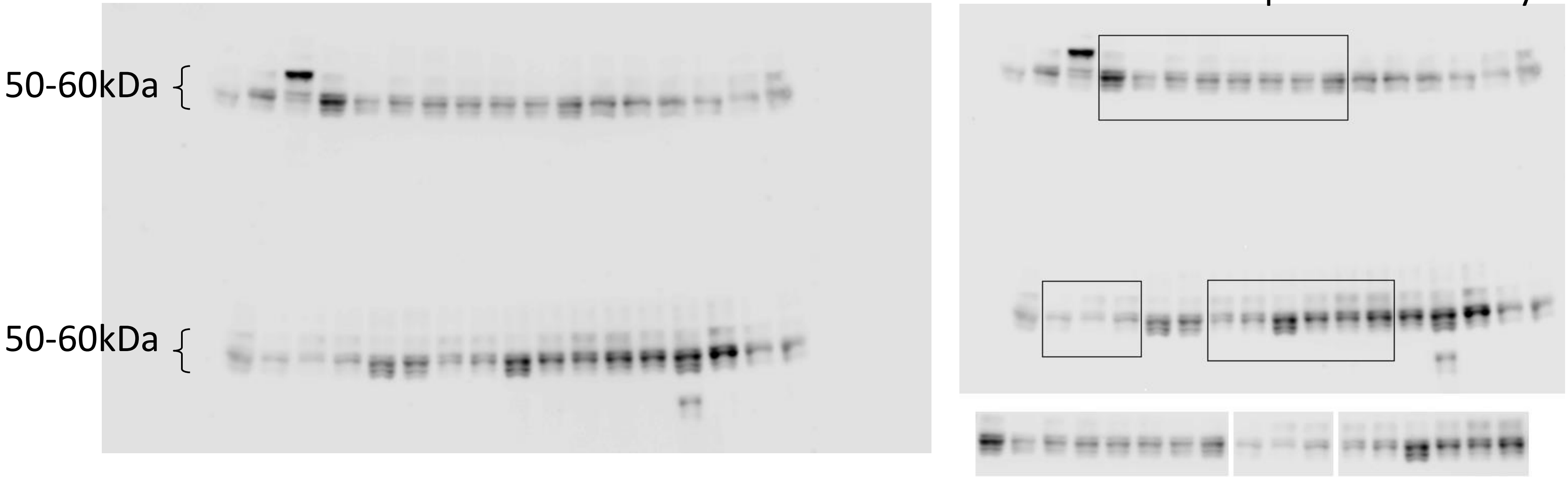

Olfactory bulb pTau (AT8)

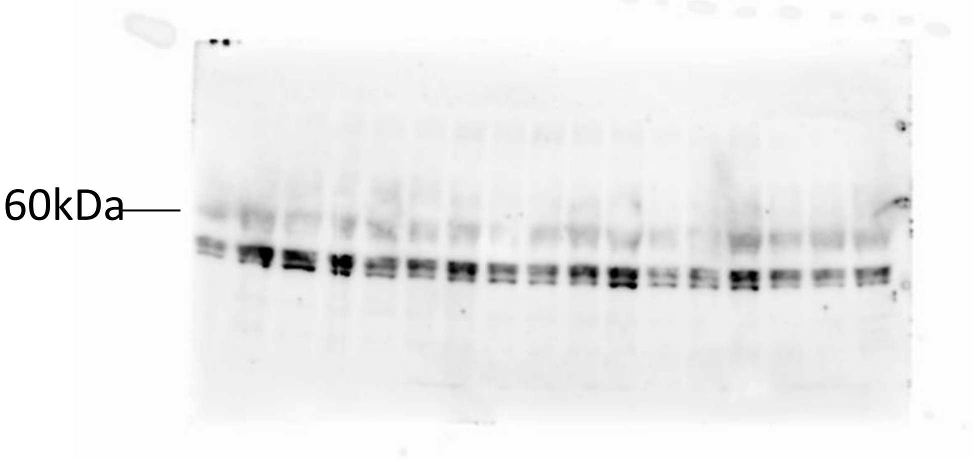

Olfactory bulb BDNF

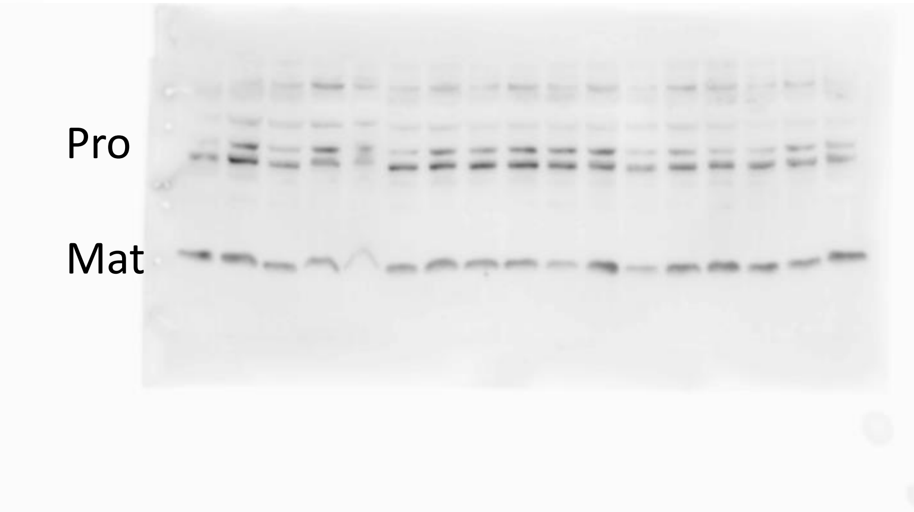

Olfactory bulb Total tau (*including other samples)

50-60kDa

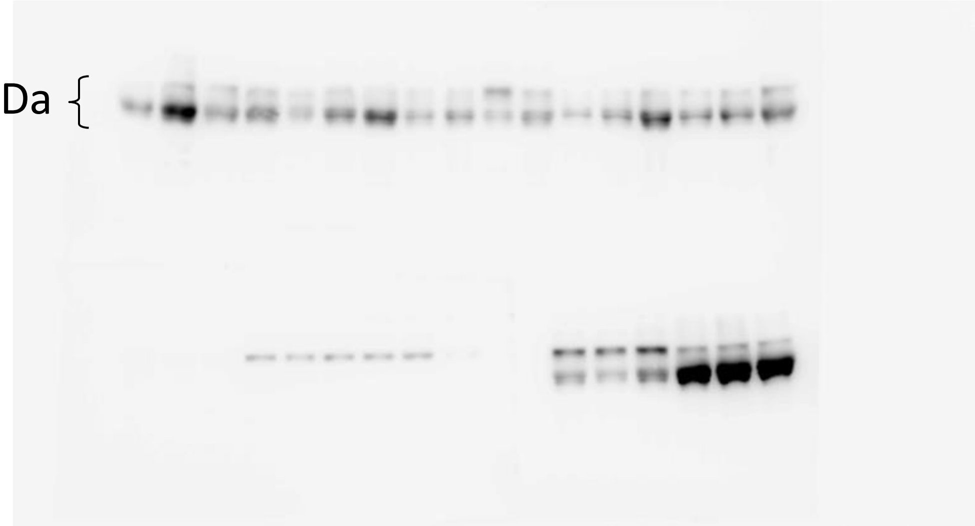

: the samples in this study

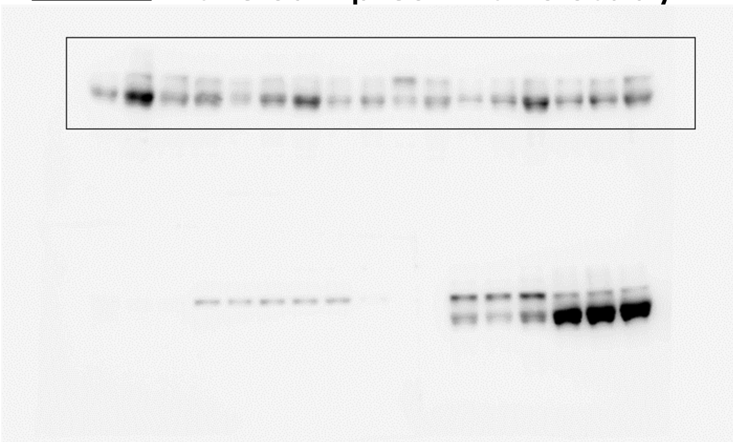

Olfactory bulb GAPDH (*including other samples, reblot after tau detection shown above)

GAPDH (37kDa)

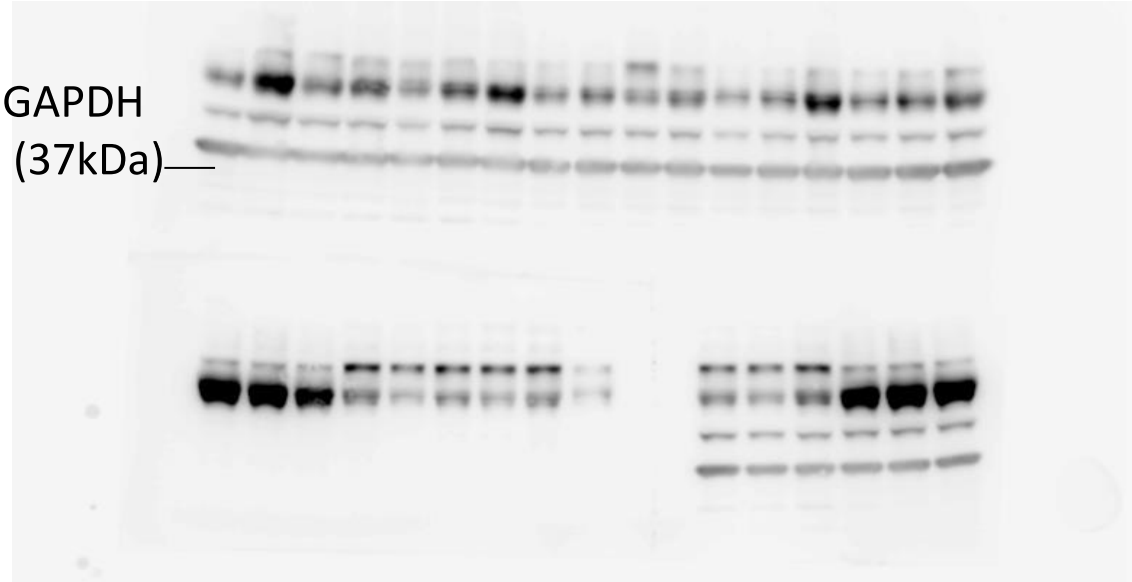

: the samples in this study

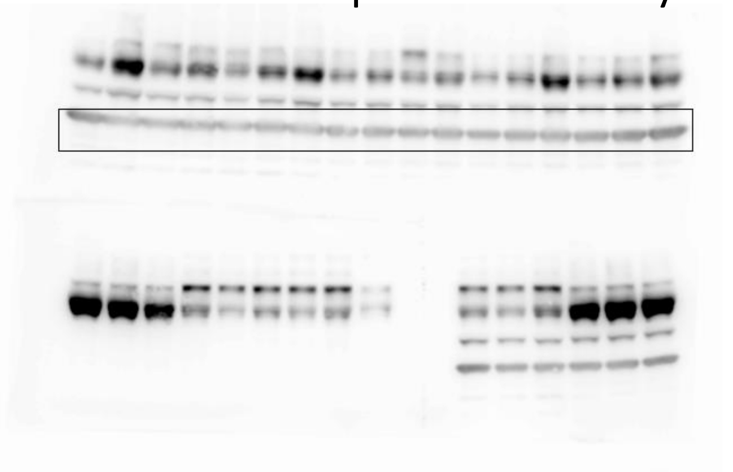

Supplement: S1 Fig — (PDF) [file pone.0240378.s001.pdf]
